# 

**Published:** 1878-10

**Authors:** 


### VIRGINIA STATE DENTAL ASSOCIATION ANNOUNCEMENT.

*To the Members of the Virginia State Dental Association and the Profession generally:*

Gentlemen:—The regular annual meeting of the Virginia State Dental Association will be held in the city of Richmond on the second Monday in December, 1868.

It is deemed highly important for the interests of the profession throughout the State, that this meeting should be as full as possible.

A cordial invitation is hereby extended, not only to the members of the society, but to all members of the profession, whether residing in this or other states, to meet with us.

Those residing in this State, and not members of the Society, are urgently invited to come and unite with us, thus lending their aid in carrying forward the enterprises which have for their end the good and welfare of the whole profession.

The members of the regular Standing Committees are earnestly urged to prepare as full and complete reports as possible, experience having shown that the more faithful the committees perform the duties assigned them, the more interesting and useful our meetings are made.

The resident executive committee is charged with the duty of providing entertainment for non-resident members and visitors during their stay in the city, and from the well known energy and enterprise of the gentlemen composing this committee, we have good reason to believe that their portion of the programme will be carried out to the satisfaction of all.

REGULAR STANDING COMMITTEES FOR THE YEAR 1878.

Committe on Publication.—Drs. L. M. Cowardin, Richmond; G. Smith, Louisa C. H.; D. B. Garden, Farmville.

Committee on Operative Dentistry.—Drs. S. H. Henkle, Staunton; John W. Scribner, Charlottsville; Wm. Farmer, Wytheville.

Committee on Mechanical Dentistry.—Drs. G. A. Sprinkle, Culpeper; Prof. J. B. Hodgkin, Baltimore, M. D.; W. E. Norris, Charlottsville.

Committee on Dental Education and Literature.—Drs. George H. Chewning, Fredericksburg; Dr. Frank L. Harris, Staunton; S. G, Cowardin, Richmond.

Committe on Dental Pathology.—Drs. N. M. Burkholder, Harrisonburg; Jud. B. Wood, Richmond; Jas. F. Thompson, Fredericksburg.

Committee on Physiology, Histology, Microscopy and Chemistry.—Drs. W. W. H. Thackston, Farmville; James Johnston, Staunton; Frank A. Jeter, Richmond.

Committee on Dental Appliances.—Drs. W. Leigh Burton, Richmond; J. O. Hodgkin, Warrentown; R. Y. Henly King & Queen.

Executive Committee.—Drs. Jud. B. Wood, Frank A. Jeter, Frank L. Harris.

J. Hall Moore,

President.

L. M. Cowarden,

Cor. Secretary.

### CONTENTS.

COMMUNICATIONS.

### *THE DENTAL REGISTER,*

A MONTHLY MAGAZINE DEVOTED TO THE INTERESTS OF

THE DENTAL PROFESSION.

*Terms Reduced to $2 50 per Year. Single Copies 25c.*

*EDITED BY PROF. J. TAFT, D. D. S.,*

AND PUBLISHED BY

SPENCER & CROCKER, Ohio Dental Depot.

The volume commences in January and closes in December.

The Register being issued monthly is always fresh, therefore Indispensable to the practitioner who desires to keep posted up with the new inventions and improvements which are daily developed. Neither expense nor effort will be spared to give value and variety to its contents. Articles will be illustrated with well executed engravings whenever they will add to the interest or assist to explanation.

Its extensive circulation and frequency of issue make it one of the BEST DENTAL ADVERTISING MEDIUMS in the United States.

All subscrptions must begin with January or July.

Local or special notices, five lines of less, will be inserted for $3.00 each insertion; over five lines, 50 cts. per line.

All advertising bills payable in advance, or at furthest, after the issue of the first number containing the advertisement. All transient advertisements to be paid for in advance.

☞ CONTRIBUTIONS SOLICITED.

Exclusively Editorial Communications should be addressed to Editor.

The latest number of the Register may be ordered with or without other goods at any time, and all letters should be addressed to

**SPENCER & CROCKER, Ohio Dental Depot, Cincinnati, O.**

